# A multicenter study to compare the effectiveness of the inpatient post acute care program versus traditional rehabilitation for stroke survivors

**DOI:** 10.1038/s41598-022-16984-9

**Published:** 2022-07-27

**Authors:** Ke-Vin Chang, Kai-Hua Chen, Ying-Hsun Chen, Wei-Chih Lien, Wei-Han Chang, Chung-Liang Lai, Cheng-Che Wu, Chia-Hsin Chen, Yu-Hsin Chen, Wei-Ting Wu, Tyng-Guey Wang, Der-Sheng Han

**Affiliations:** 1 Department of Physical Medicine and Rehabilitation, National Taiwan University Hospital and National Taiwan University College of Medicine, No. 7, Chung Shan S. Rd., Zhongzheng Dist., Taipei, 100 Taiwan; 2 Department of Physical Medicine and Rehabilitation, National Taiwan University Hospital, Bei-Hu Branch, No. 87, Neijiang Street., Wanhua District, Taipei, 108 Taiwan; 3 Center for Regional Anesthesia and Pain Medicine, Taipei Municipal Wang-Fang Hospital (Managed by Taipei Medical University), Taipei, Taiwan; 4 Department of Physical Medicine and Rehabilitation, Chang Gung Memorial Hospital, Chiayi, Taiwan; 5 School of Medicine, College of Medicine, Chang Gung University, Taoyüan, Taiwan; 6 Graduate Institute of Education, National Chung Cheng University, Chiayi, Taiwan; 7 Department of Rehabilitation Medicine, Taipei City Hospital, Renai Branch, Taipei, Taiwan; 8 Department of Physical Medicine and Rehabilitation, National Cheng Kung University Hospital, College of Medicine, National Cheng Kung University, Tainan, Taiwan; 9 Department of Physical Medicine and Rehabilitation, College of Medicine, National Cheng Kung University, Tainan, Taiwan; 10 Department of Physical Medicine and Rehabilitation, Taoyuan Chang Gung Memorial Hospital, Taoyüan, Taiwan; 11 Department of Physical Medicine and Rehabilitation, Keelung Chang Gung Memorial Hospital, Keelung, Taiwan; 12 Department of Physical Medicine and Rehabilitation, Puzi Hospital, Ministry of Health and Welfare, Chiayi, Taiwan; 13 Department of Occupational Therapy, Asia University, Taichung, Taiwan; 14 Department of Physical Medicine and Rehabilitation, Sinwu Branch, Taoyuan General Hospital, Ministry of Health and Welfare, Taoyüan, Taiwan; 15 School of Post-Baccalaureate Medicine, College of Medicine, Kaohsiung Medical University Hospital, Kaohsiung, Taiwan; 16 Department of Physical Medicine and Rehabilitation, Kaohsiung Medical University Chung-Ho Memorial Hospital, Kaohsiung, Taiwan

**Keywords:** Cardiology, Health care, Neurology

## Abstract

There is insufficient evidence to prove the effect of the Post-acute Care (PAC) program on post-stroke recovery. This study aimed to determine the effectiveness of the PAC versus traditional inpatient rehabilitation (non-PAC) for middle- and old-aged stroke survivors. This multicenter cohort study enrolled 334 stroke patients admitted for post-stroke rehabilitation. The outcome variables included the Barthel Index (BI), Functional Oral Intake Scale (FOIS), Mini Nutritional Assessment-Short Form (MNA-SF), EuroQoL-5D (EQ-5D), Lawton–Brody Instrumental Activities of Daily Living (ADL) Scale, and Mini-Mental State Examination (MMSE). The inverse-probability-of-treatment-weighting method was used to analyze the differences in outcomes between the PAC and non-PAC groups. The PAC group showed better improvements in BI, MNA-SF, EQ-5D, Instrumental ADL, and MMSE compared to the non-PAC group, with differences in effect sizes of 0.54 (95% confidence interval [CI] 0.38–0.71), 0.26 (95% CI 0.10–0.42), 0.50 (95% CI 0.33–0.66), 0.44 (95% CI 0.28–0.60) and 0.34 (95% CI 0.17–0.50), respectively. The PAC project showed more improvement in basic and instrumental ADL and status of swallowing, nutrition, and cognition than those of non-PAC, which had less length of stay restricted by the National Health Insurance. More studies are warranted to investigate the influence of hospital stay and duration from stroke onset on the PAC’s effectiveness.

## Introduction

The Global Burden of Diseases, Injuries, and Risk Factors Study in 2019 showed that stroke is the second leading cause of disability-adjusted life-years above 50 years, after ischemic heart disease^1^. Stroke survivors often experience multidimensional impairment, encompassing limb weakness, ataxia, dysphagia, and aphasia. The suggested golden period of post-stroke rehabilitation is within 3 months after the cerebral vascular event, during which 48–91% of recovery takes place^2^. Therefore, early rehabilitation with the integration of physical, occupational, and speech therapies should be arranged for stable patients to facilitate the restoration of physical functions and reduce the impact of post-stroke sequelae^3^. Intensive rehabilitation during the early post-stroke stage usually requires hospitalization to ensure hemodynamic stability and prevent the evolution of neurological deficits.

Restricted by the National Health Insurance (NHI) policy, the traditional inpatient post-stroke rehabilitation has several disadvantages in Taiwan. First, the admission criteria are at the discretion of attending physician, without a standard evaluation process. Second, the patients or their families must find a suitable rehabilitation unit after discharge from the acute-care ward, which is time-consuming and inefficient. Third, the copayment for traditional inpatient rehabilitation is relatively low in Taiwan, which subsequently leads to a compensatory reduction in training amount and intensity. In 2014, the NHI Administration in Taiwan initiated the Post-acute Care (PAC) program targeting cerebrovascular disease^4,5^. Following the acute phase, stroke survivors who met the admission criteria were transferred to the authorized hospitals without disruption of the inward care, where custom-made comprehensive rehabilitation was provided by a multidisciplinary team for a maximal length of stay of 12 weeks. The participants of both the PAC and non-PAC groups accepted the same quality of rehabilitation assessment and training. In Taiwan, the physiatrists are mandatory to receive the consultation, regularly assess the functional status of the participants, and give the rehabilitation prescriptions, even the PAC program was performed in non-rehabilitation ward. Several studies showed that the PAC program reduced hospital stay and associated medical costs^6,7^. Despite the positive outcomes of the PAC program in alleviating economic burdens on the medical care system, the evidence demonstrating its effect on post-stroke functional recovery remains insufficient. Therefore, the present study aimed to determine whether the PAC program was comparable or even superior to traditional inpatient rehabilitation (non-PAC) regarding functional improvement among middle- and old-aged stroke survivors.

## Results

**Figure 1:**
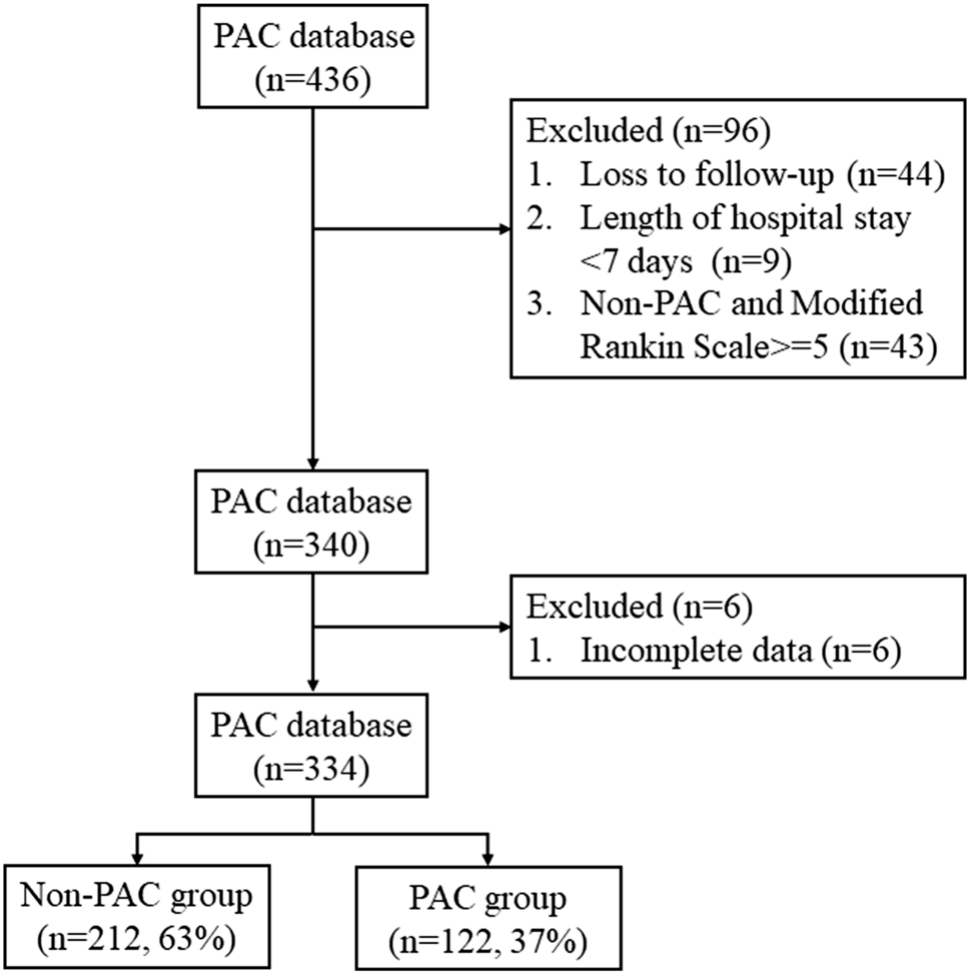
Flowchart of recruitment.

This study enrolled a total of 436 patients. After excluding 44 patients without data on the second follow-up, 9 with lengths of hospital stays < 7 days, 43 with modified Rankin Scale (MRS) scores of ≥ 5 upon admission and 6 with incomplete data, the final analysis included 334 patients, including 212 (63%) in the non-PAC group and 122 (37%) in the PAC group (Fig. 1).

Before using the inverse probablity of treatment weighting (IPTW) approach, the PAC group had higher age, longer length of hospital stay, lower Mini Nutritional Assessment-Short Form (MNA-SF) and Instrumental Activities of Daily Living (IADL) scores, higher EuroQoL-5D (EQ-5D) score, and a greater proportion of ischemic stroke. After IPTW, no significant differences were identified in most of the variables except for the length of hospital stay and IADL (Table 1).

**Table 1:** The characteristics of the patients before and after being weighted by using the inverse probability of treatment weighting method.

	Unweighted		*p* value	Weighted		*p* value
	Non-PAC	PAC		Non-PAC	PAC	
	(n = 212)	(n = 122)				
Age, years	62.07 ± 13.95	66.38 ± 13.95	0.007*	63.31 ± 13.75	64.14 ± 14.82	0.616
Male	70.3%	64.8%	0.296	68.8%	70.1%	0.704
BMI, kg/m^2^	24.82 ± 3.85	24.96 ± 4.45	0.764	24.84 ± 3.88	24.82 ± 4.56	0.968
Length of hospital stay, days	27.32 ± 12.87	48.73 ± 28.00	< 0.001*	26.24 ± 12.24	53.39 ± 27.68	< 0.001*
**Functional score at admission**						
MRS	3.82 ± 0.45	3.75 ± 0.44	0.164	3.82 ± 0.44	3.77 ± 0.42	0.301
BI	36.7 ± 23.91	38.4 ± 24.08	0.532	37.11 ± 23.55	37.06 ± 23.96	0.987
FOIS	5.78 ± 2.07	5.93 ± 1.91	0.532	5.81 ± 2.04	5.84 ± 1.93	0.872
MNA-SF	10.86 ± 2.11	10.04 ± 1.80	< 0.001*	10.67 ± 2.20	10.38 ± 1.57	0.165
EQ-5D	0.03 ± 0.32	0.15 ± 0.35	< 0.001*	0.05 ± 0.32	0.02 ± 0.38	0.486
IADL	4.85 ± 2.97	3.23 ± 2.02	< 0.001*	4.33 ± 2.96	3.60 ± 2.03	0.010*
MMSE	19.81 ± 9.58	20.13 ± 8.98	0.761	20.02 ± 9.36	20.50 ± 9.06	0.636
**Comorbidities**						
Diabetes mellitus	34.0%	27.9%	0.249	32.7%	34.0%	0.738
Hypertension	65.1%	63.9%	0.831	67.5%	72.2%	0.193
Atrial fibrillation	11.8%	10.7%	0.753	12.6%	11.5%	0.643
Heart disease	10.4%	9.8%	0.875	10.1%	6.9%	0.151
**Type of stroke**						
Ischemic stroke	11.8%	24.6%	0.002*	17.0%	20.5%	0.245
Hemorrhagic stroke	7.1%	12.3%	0.108	8.1%	8.2%	0.961
**Areas of stroke**						
Left Hemisphere	42.9%	33.6%	0.094	39.4%	38.0%	0.719
Right Hemisphere	41.5%	34.4%	0.201	41.7%	41.8%	0.984
Cerebellar	2.8%	1.6%	0.715	2.1%	0.8%	0.125
Brain Stem	7.1%	5.7%	0.635	7.8%	8.6%	0.693
Other	7.1%	1.6%	0.030*	8.6%	0.6%	< 0.001*

Data are presented as mean ± standard deviation or %

*SD* standard deviation, *PAC* post-acute care, *BMI* body mass index, *MRS* modified Rankin scale, *BI* Barthel index, *FOIS* functional oral intake scale, *MNA-SF* mini nutrition assessment-short form. *EQ-5D* EuroQoL-5D, *IADL* Lawton–Brody instrumental activities of daily living scale, *MMSE* mini-mental state exam.

*p value < 0.05.

Before IPTW, both groups showed significant increases in the scores of the primary (Barthel index) and secondary (functional oral intake scale [FOIS], MNA-SF, EQ-5D, IADL, and mini-mental state exam [MMSE]) outcomes compared to the values at admission (Table 2). The improvements remained consistent after the data had been weighted (Table 3).

**Table 2:** Unweighted total score for each functional status measure before and after admission in the non-PAC and PAC groups.

Measures	Non-PAC		Difference (after-before) (95% CI)	*p* value	PAC		Difference (after-before) (95% CI)	*p* value
	(n = 212)				(n = 122)			
	Before	After			Before	After		
	Mean ± SD	Mean ± SD			Mean ± SD	Mean ± SD		
**Primary outcome**								
BI	36.70 ± 23.91	52.57 ± 25.91	15.87 (13.30–18.44)	< 0.001*	38.40 ± 24.08	64.18 ± 25.29	25.78 (22.17–29.39)	< 0.001*
**Secondary outcome**								
FOIS	5.78 ± 2.07	6.36 ± 1.45	0.58 (0.37–0.78)	< 0.001*	5.93 ± 1.91	6.65 ± 1.14	0.72 (0.41–1.04)	< 0.001*
MNA-SF	10.86 ± 2.11	11.53 ± 1.95	0.67 (0.46–0.87)	< 0.001*	10.04 ± 1.80	11.16 ± 1.75	1.11 (0.80–1.42)	< 0.001*
EuroQoL-5D	0.03 ± 0.32	0.20 ± 0.32	0.17 (0.14–0.21)	< 0.001*	0.15 ± 0.35	0.38 ± 0.32	0.23 (0.17–0.29)	< 0.001*
IADL	4.85 ± 2.97	4.96 ± 2.84	0.11 (− 0.09–0.32)	0.270	3.23 ± 2.02	4.43 ± 2.47	1.20 (0.86–1.51)	< 0.001*
MMSE	19.81 ± 9.58	22.02 ± 9.25	2.13 (1.57–2.69)	< 0.001*	20.13 ± 8.98	23.50 ± 7.92	3.37 (2.52–4.21)	< 0.001*

*SD* standard deviation, *CI* confidence interval, *PAC* post-acute care, *Non-PAC* traditional inpatient rehabilitation, *BI* Barthel index, *FOIS* functional oral intake scale, *MNA-SF* mini nutrition assessment-short form, *EQ-5D* EuroQoL-5D, *IADL* Lawton–Brody instrumental activities of daily living scale, *MMSE* mini-mental state examination.

*p value < 0.05.

**Table 3:** Weighted total score for functional status measures before and after admission in the non-PAC and PAC groups.

Measures	Non-PAC		Difference (after-before) (95% CI)	*p* value	PAC		Difference (after-before) (95% CI)	*p* value
	(n = 212)				(n = 122)			
	Before	After			Before	After		
	Mean ± SD	Mean ± SD			Mean ± SD	Mean ± SD		
**Primary outcome**								
BI	37.11 ± 23.55	52.52 ± 26.03	15.41 (12.94–17.89)	< 0.001*	37.06 ± 23.96	68.07 ± 27.47	31.0 (26.97–35.05)	< 0.001*
**Secondary outcome**								
FOIS	5.81 ± 2.04	6.33 ± 1.46	0.52 (0.33–0.71)	< 0.001*	5.84 ± 1.93	6.69 ± 1.07	0.84 (0.52–1.16)	< 0.001*
MNA-SF	10.67 ± 2.20	11.43 ± 2.00	0.76 (0.55–0.97)	< 0.001*	10.38 ± 1.57	11.74 ± 1.73	1.36 (1.05–1.67)	< 0.001*
EuroQoL-5D	0.05 ± 0.32	0.21 ± 0.31	0.16 (0.13–0.20)	< 0.001*	0.02 ± 0.38	0.40 ± 0.34	0.38 (0.31–0.44)	< 0.001*
IADL	4.33 ± 2.96	4.59 ± 2.83	0.27 (0.06–0.48)	0.012*	3.60 ± 2.03	4.86 ± 2.51	1.26 (0.95–1.56)	< 0.001*
MMSE	20.02 ± 9.36	22.02 ± 8.99	1.94 (1.41–2.47)	< 0.001*	20.50 ± 9.06	24.57 ± 7.83	4.07 (3.18–4.97)	< 0.001*

*SD* standard deviation, *CI* confidence interval, *PAC* post-acute care, *BI* Barthel index, *FOIS* functional oral intake scale, *MNA-SF* mini nutrition assessment-short form, *EQ-5D* EuroQoL-5D, *IADL* Lawton–Brody instrumental activities of daily living scale, *MMSE* mini-mental state examination.

*p value < 0.05.

Compared to the non-PAC group, the PAC group showed better improvements in BI, MNA-SF, EQ-5D, IADL, and MMSE, with difference in the effect sizes of 0.54 (95% confidence interval [CI] 0.38–0.71), 0.26 (95% CI 0.10–0.42), 0.50 (95% CI 0.33–0.66), 0.44 (95% CI 0.28–0.60), and 0.34 (95% CI 0.17–0.50), respectively. A similar trend with borderline significance was observed for FOIS, with a difference in the effect size of 0.14 (95% CI − 0.01–0.3) (Table 4).

**Table 4:** Difference in effect size in each functional status measure before and after admission between the PAC and non-PAC groups.

Measures	Non-PAC		ES of after admission versus before	PAC		ES of after admission versus before	Mean difference in ES of PAC versus Non-PAC (95% CI)
	(n = 212)			(n = 122)			
	Before	After		Before	After		
	Mean ± SD	Mean ± SD		Mean ± SD	Mean ± SD		
**Primary outcome**							
BI	37.11 ± 23.55	52.52 ± 26.03	0.54	37.06 ± 23.96	68.07 ± 27.47	1.08	0.54 (0.38–0.71) *
**Secondary outcome**							
FOIS	5.81 ± 2.04	6.33 ± 1.46	0.23	5.84 ± 1.93	6.69 ± 1.07	0.37	0.14 (− 0.01–0.30)
MNA-SF	10.67 ± 2.20	11.43 ± 2.00	0.33	10.38 ± 1.57	11.74 ± 1.73	0.59	0.26 (0.10–0.42) *
EQ-5D	0.05 ± 0.32	0.21 ± 0.31	0.36	0.02 ± 0.38	0.40 ± 0.34	0.86	0.50 (0.33–0.66) *
IADL	4.33 ± 2.96	4.59 ± 2.83	0.12	3.60 ± 2.03	4.86 ± 2.51	0.56	0.44 (0.28–0.60) *
MMSE	20.02 ± 9.36	22.02 ± 8.99	0.31	20.50 ± 9.06	24.57 ± 7.83	0.65	0.34 (0.17–0.50) *

*SD* standard deviation, *CI* confidence interval, *ES* effect size, *PAC* post-acute care, *BI* Barthel index, *FOIS* functional oral intake scale, *MNA-SF* mini nutrition assessment-short form, *EQ-5D* EuroQoL-5D, *IADL* Lawton–Brody instrumental activities of daily living scale, *MMSE* mini-mental state exam.

*p value < 0.05.

## Discussion

This study yielded two important findings. First, both PAC and non-PAC groups showed significant increases in the primary (BI) and secondary (FOIS, MNA-SF, EQ-5D, IADL, and MMSE) outcomes compared to the values upon admission. Second, significantly more improvement was observed in the PAC group than in the non-PAC group in nearly all assessed outcome variables.

A multicenter study of 849,780 adults with stroke in the United States (US) reported that most patients (56.4%) were discharged to the PAC service^8^. There was also an increase in PAC utilization from 2003 to 2011. This survey also observed a greater increase in discharge to inpatient rehabilitation facilities than to skilled nursing facilities and home health providers. Another US cohort study with a large national sample of 99,185 people with stroke demonstrated that inpatient rehabilitation was associated with greater improvement in physical function and health care skills compared to rehabilitation in skilled nursing facilities^9^. In Taiwan, all PAC services were provided in accredited hospitals, which was considered the most effective type of functional recovery for stroke survivors. In 2017, Lai et al. reported that 76.8% of stroke patients returned to their homes and communities following the completion of the PAC program in Taiwan^10^. Several factors are associated with the outcomes of patients utilizing the post-stroke PAC program, including the duration of PAC stay^11^ and stroke type^12^. However, most hospitals authorized to provide the PAC program in Taiwan also have non-PAC services. The spaces, facilities, and therapists allocated to the patients using the PAC program usually overlapped with those admitted to the non-PAC program. Therefore, comparisons of the effectiveness of functional recovery between the PAC and non-PAC programs are paramount for policymakers to determine the resource allocation for post-acute stroke inpatient care.

Both the PAC and non-PAC groups exhibited significant improvements in all outcome variables after inpatient rehabilitation. In our study, regardless of which group the participants were assigned to, structured inpatient rehabilitation was provided despite the differences in the treatment intensity and duration. Overall, our findings were consistent with those reported in a cohort of 722 patients admitted for inpatient rehabilitation within 90 days of stroke onset, showing a positive gain in functional independence at discharge^13^. That study also identified several prognostic factors, such as age, marital status, and presence of aphasia. Because the basic demographics (such as age) were not similar across our two study groups, analysis using the IPTW method would help to clarify the between-group differences in outcome improvement.

Our data revealed that the PAC group showed more improvement in BI, MNA-SF, EQ-5D, IADL, and MMSE scores compared to those in the non-PAC group. In 2021, Chiu et al. analyzed 910 stroke patients using propensity score matching for comparisons between PAC and non-PAC programs^7^. They showed that the PAC group had significantly more functional gain in BI, FOIS, EQ-5D, IADL, and MMSE scores compared to the non-PAC group, consistent with our findings. We observed that the effect sizes of the between-group differences mostly exceeded 0.2 (small to moderate effect)^14^ in most outcome variables such as BI, MNA-SF, EQ-5D, IADL, and MMSE. Thus, compared to traditional inpatient rehabilitation, the PAC program provided additional clinically important benefits to patients’ ability to perform basic and instrumental self-care, as well as their nutritional status and quality of life.

The superiority in clinical outcomes of the PAC program over the non-PAC program could be attributed to several factors. First, the length of stay in the PAC group was longer than that in the non-PAC group. Patients admitted to the PAC program could extend their admission period to a maximum of 12 weeks if the potential for function recovery was recognized by the consensus of the rehabilitation team members. In contrast, the maximal duration of admission in the non-PAC program depended heavily on the decision of the physicians in charge as well as the policy of each hospital, which was usually less than 1 month to prevent rejection of reimbursement by the insurance supplier. Furthermore, patients who participated in the non-PAC program might be transferred from one hospital to another until the designated goal was achieved and the treatment discontinuity would possibly cause temporary functional decline. As the extended length of hospital stay is also a core component of the PAC program, it is hard to separate this factor from efficacy analysis.

A previous clinical trial examining the effectiveness of prolonged inpatient rehabilitation in 52 patients with subacute stroke^15^ reported significantly more improvement in the modified motor assessment scale, timed up and go test, Berg balance scale, BI, and 36-item short-form survey at 6 months post-intervention in the experimental group compared to the control group. These results, along with those of the present study, implied the benefits of extended inpatient rehabilitation (up to 3 months) in improving activities of daily living, mobility, and quality of life in stroke survivors.

Second, the PAC project provided a higher amount of rehabilitation training compared to the non-PAC program. A cohort study enrolling 123 patients admitted with subacute stroke^16^ identified significant positive associations between Functional Independence Measure scores and the total time spent on physical and occupational therapy. In our study, the overall periods of daily rehabilitation ranged from three to five sessions (50 min each) in the PAC program under the rule of reimbursement per diem. However, the maximum allowance of rehabilitation training in the non-PAC program was three sessions per day, based on the regulation of our national insurance bureau. Therefore, the authorization of extended durations in inpatient rehabilitation every day was another likely explanation for the better outcome in the PAC group.

Our study had several strengths: First, the IPTW method was used instead of a propensity score-matching method^17^, which allowed the inclusion of all eligible patients to investigate the treatment effect. Second, we excluded from our analysis patients in the non-PAC group with an MRS of ≥ 5, as the PAC group only enrolled patients with an MRS of 2–4. This approach could improve the comparability between the PAC and non-PAC groups to minimize the influence of baseline discrepancy on our outcome estimations. Third, our data were collected from multiple hospitals in different counties in Taiwan. The multi-center design improved the generalizability of our results.

The present study had several limitations. First, a randomized controlled design was not used and there was a discrepancy in the basic characteristics between the PAC and non-PAC groups. Future randomized controlled trials are needed to investigate whether certain confounders at baseline could cause overestimation of the effect of the PAC program. Second, the patients included in the PAC program were within 40 days of stroke onset. Therefore, we were unable to determine whether the benefits of the PAC program for stroke patients persisted in the subacute stage, which requires further investigation. Third, since some PAC programs in Taiwan are not provided in the rehabilitation ward led by physiatrists: eg. nursing home- or home-based PAC, whether the training effectiveness of these non-rehabilitation ward-based PACs are the same merits further clinical trials. Fourth, the efficacy divided by the length of stay seems to be plausible for analyzing the unbalanced length of stay between the PAC and non-PAC groups. However, according to the systematic review authored by Hatem et al.^18^, the post-stroke neurological recovery presented with a nonlinear, logarithmic pattern. The greatest recovery was mostly identified within the 3 months following stroke. Due to the non-linear functional improvement, the approach of efficacy per day might not be suitable for outcome analysis. Fifth, the patients in the PAC program might stay longer in the same hospital than those in the non-PAC program. However, the patients in the non-PAC program would be admitted from one hospital to another if their improvement did not meet the expectation. In our study, we did not have the data of the exact length of stay of the whole hospitalization process. Therefore, the comparison of the total cost between the PAC and non-PAC programs could not be conducted through this study. Sixth, the patients in the study were assigned either to the PAC or non-PAC program, each of which was incorporated with intervention of rehabilitation. Therefore, using the current research framework, we were unable to know the effectiveness of the PAC or non-PAC program in comparison with the nature recovery course after stroke. Seventh, we did not have data regarding the disposition of the patients after completion of the PAC or non-PAC program. Empirically, most of the patients in the PAC program returned home due to better functional improvement. On the other hand, a substantial portion of the patients in the non-PAC program would be transferred to another hospital for inpatient rehabilitation before meeting the designated goal. However, the above statement was based on our crude observation but not on strict statistical analyses. Therefore, more prospective studies are needed to investigate this issue.

## Methods

### Study design

This multicenter cohort study involved seven teaching and two community hospitals in Taiwan. The primary investigators of each hospital were all physiatrists who oversaw responses to patient referrals and subsequent assignments. Patients who joined the PAC program were referred from the upper-stream medical centers or teaching hospitals, whereas those in the non-PAC course only required the agreement of the attending physicians for admission. All patients were required to (1) be older than 18 years, (2) have a diagnosis of stroke confirmed by clinical presentations and imaging findings.

This study was approved by the Research Ethics Committee Office of National Taiwan University Hospital (No. 201803013RINA) and the Institutional Review Board (IRB) of Chang Gung Memorial Hospital (IRB No. 201800767B0C501). The informed consent was obtained from all subjects involved in the study. The informed consent was obtained from all subjects involved in the study. All methods were carried out in accordance with relevant guidelines and regulations (the Declaration of Helsinki).

### Participants

The initial criterion for admission to the PAC program was 30 days following a stroke episode^6,10^. In 2017, the criteria were changed to acute stroke within 40 days after onset with a MRS score between 2 and 4. The MRS is commonly used to assess the degree of disability, with a higher score indicating more dependence on daily activities. The MRS level ranges from 0 (no symptoms) to 6 (death)^19^.

Among the patients who participated in the non-PAC program, there was no strict regulation for the range of pre-admission MRS. The admission criteria were (1) stroke onset within 1 year before admission, (2) sufficient cognition to comprehend the instructions, and (3) endurance expected to tolerate rehabilitation exercises.

### Intervention

#### PAC program

Rehabilitation in the PAC program was provided by a multidisciplinary team comprising physiatrists, physical therapists, occupational therapists, speech therapists, psychologists, social workers, and nurses. The prescriptions for therapeutic items were provided by the physiatrists after comprehensive evaluations of the patients, who underwent at least three sessions of therapy per weekday. Each session lasted for 50 min and was directed by physical, occupational, or speech therapists. The rehabilitation program was dynamically tailored according to the progress of the patient’s condition and incorporated passive range of motion exercise, facilitation, therapeutic exercise, muscle strengthening, balance training, transfer, upper and lower extremity function and activities of daily living, speech therapy, and swallowing training. The principal period of the PAC program was 3–6 weeks and the service providers were entitled to apply for an extension for up to 12 weeks based on the patient’s progress^4,6,10^.

#### Non-PAC program

In contrast to the PAC program, the eligibility for the non-PAC program was more subjective, mainly relying on the perception of the attending physician of the patient’s potential for functional recovery. The length of the non-PAC program was restricted by the NHI policy, mostly less than 1 month. The rehabilitation service consisted of one session of physical therapy and one session of occupational therapy per weekday. Patients with aphasia or dysphagia were entitled to receive 2–3 speech therapy sessions per week. Each session lasted for no more than 50 min. Unlike the PAC program, a regular interdisciplinary group meeting to exchange expert opinions for the patient’s progress was not mandatory for the non-PAC program.

### Primary outcome

#### BI

The BI is an ordinal scale designed to evaluate the performance of activities of daily living and was defined as the primary outcome in this study. A total of ten activities were assessed, including feeding, grooming, toileting, bathing, dressing/undressing, bladder control, bowel control, transfer between the wheelchair and bed, and ability to walk or propel a wheelchair. The maximum BI score is 100 points, with a higher value indicating better functional independence^20^.

### Secondary outcomes

#### FOIS

The FOIS is a 7-point ordinal scale used as a surrogate measurement for dysphagia. The level of swallowing impairment is rated based on the route of oral intake and food amount and consistency, grading from nothing by mouth to total oral diet without restrictions. A higher summary score indicates better swallowing function^21^. Patients with an FOIS ≤ 5 were considered to have dysphagia and swallowing training would be given regardless of which group they had to been assigned to.

#### MNA-SF

The MNA-SF test has six items, including anthropometric measurements (body mass index, weight loss), global assessment (mobility), and dietary questionnaire and subjective assessment (food intake, neuropsychological problems, acute disease). Overall scores of < 8, 8–11, and > 11 indicate malnutrition, risk of malnutrition, and no malnutrition, respectively^22^.

#### EQ-5D

The EQ-5D is a well-known instrument used to evaluate patient quality of life. It comprises five items: mobility, self-care, pain, usual activities, and psychological status, each of which is rated at three levels: 1 (no problem), 2 (moderate problem), and 3 (severe problem)^23^.

#### IADL scale

The IADL scale comprises eight items, each of which is scored 1 point, for the evaluation of the patient’s ability to use the telephone, shop, prepare food, perform housekeeping tasks, clean laundry, transport self, prepare own medication, and handle finances. The summary score ranges from 0 (lowest function) to 8 (highest function)^24^.

#### MMSE

The MMSE, a 30-point questionnaire, is widely used to evaluate cognitive function. A total of nine categories are assessed, including orientation to time, orientation to place, registration, attenuation/calculation, recall, language repetition, and complex commands. A summary score of ≥ 24 indicates normal cognition, whereas scores of 19–23, 10–18, and ≤ 9 indicate mild, moderate, and severe cognitive impairment, respectively^25^.

### Statistical analysis

The IPTW method was used to analyze the outcome differences between the PAC and non-PAC groups^17^. Unlike the propensity matching approach, the sample size of the original data can be maintained by using the IPWT method to provide a more precise estimation of the treatment effect. Employing the IPTW method, weights were assigned to the patients according to the inverse of their probability of undergoing certain treatments (PAC versus non-PAC), which was estimated using the propensity score^17^. The propensity score was derived from a multivariate logistic regression model that included variables of patient demographics (age, sex, and body mass index), clinical characteristics (stroke type, hemiplegic side, and comorbidities), chronic disease (diabetes mellitus, hypertension, atrial fibrillation, heart disease), and pre-rehabilitation functional status (BI, FOIS, MNA-SF, EQ-5D, IADL, and MMSE).

To examine the balance of the distributions between the PAC and non-PAC groups, Student’s t- and chi-squared tests were used to compare continuous and categorical variables in the unweighted and weighted cohorts. Paired t-tests were used to compare data from repeated measurements. Shapiro–Wilk tests were used to examine the normality of the variable distributions. If the data were not normally distributed, non-parametric tests such as Mann–Whitney U and Wilcoxon signed-rank tests were used instead. Comparisons of the treatment effects were conducted using the effect sizes in the differences in functional outcome recovery. The effect sizes were calculated by dividing the mean difference by the pooled standard deviation^14^. Effect sizes of 0.2, 0.5, and 0.8, were defined as small, medium, and large differences, respectively^14^. All analyses were performed using SAS version 9.4 (SAS Institute Inc., Cary, North Carolina), and *p* < 0.05, was considered statistically significant.

## Conclusion

Traditional inpatient post-stroke rehabilitation and PAC programs are both helpful for post-stroke functional recovery. Compared to traditional inpatient rehabilitation, the PAC program showed significantly more improvement in basic and instrumental activities of daily living, nutrition, quality of life, and cognition. More prospective studies are warranted to investigate the influence of hospital stay and duration from stroke onset on the clinical effectiveness of the PAC program.

## Data Availability

The datasets generated during and analyzed during the current study are available from the corresponding author on reasonable request.
