# Author Correction: A multicenter study to compare the effectiveness of the inpatient post acute care program versus traditional rehabilitation for stroke survivors

**DOI:** 10.1038/s41598-022-18435-x

**Published:** 2022-08-18

**Authors:** Ke-Vin Chang, Kai-Hua Chen, Ying-Hsun Chen, Wei-Chih Lien, Wei-Han Chang, Chung-Liang Lai, Cheng-Che Wu, Chia-Hsin Chen, Yu-Hsin Chen, Wei-Ting Wu, Tyng-Guey Wang, Der-Sheng Han

**Affiliations:** 1 Department of Physical Medicine and Rehabilitation, National Taiwan University Hospital and National Taiwan University College of Medicine, No. 7, Chung Shan S. Rd., Zhongzheng Dist., Taipei, 100 Taiwan; 2 Department of Physical Medicine and Rehabilitation, National Taiwan University Hospital, Bei-Hu Branch, No. 87, Neijiang Street., Wanhua District, Taipei, 108 Taiwan; 3 Center for Regional Anesthesia and Pain Medicine, Taipei Municipal Wang-Fang Hospital (Managed By Taipei Medical University), Taipei, Taiwan; 4 Department of Physical Medicine and Rehabilitation, Chang Gung Memorial Hospital, Chiayi, Taiwan; 5 School of Medicine, College of Medicine, Chang Gung University, Taoyüan, Taiwan; 6 Graduate Institute of Education, National Chung Cheng University, Chiayi, Taiwan; 7 Department of Rehabilitation Medicine, Taipei City Hospital, Renai Branch, Taipei, Taiwan; 8 Department of Physical Medicine and Rehabilitation, National Cheng Kung University Hospital, College of Medicine, National Cheng Kung University, Tainan, Taiwan; 9 Department of Physical Medicine and Rehabilitation, College of Medicine, National Cheng Kung University, Tainan, Taiwan; 10 Department of Physical Medicine and Rehabilitation, Taoyuan Chang Gung Memorial Hospital, Taoyüan, Taiwan; 11 Department of Physical Medicine and Rehabilitation, Keelung Chang Gung Memorial Hospital, Keelung, Taiwan; 12 Department of Physical Medicine and Rehabilitation, Puzi Hospital, Ministry of Health and Welfare, Chiayi, Taiwan; 13 Department of Occupational Therapy, Asia University, Taichung, Taiwan; 14 Department of Physical Medicine and Rehabilitation, Sinwu Branch, Taoyuan General Hospital, Ministry of Health and Welfare, Taoyüan, Taiwan; 15 School of Post-Baccalaureate Medicine, College of Medicine, Kaohsiung Medical University Hospital, Kaohsiung, Taiwan; 16 Department of Physical Medicine and Rehabilitation, Kaohsiung Medical University Chung-Ho Memorial Hospital, Kaohsiung, Taiwan

Correction to: *Scientific Reports* https://doi.org/10.1038/s41598-022-16984-9, published online 27 July 2022

The original version of this Article contained an error in the Methods section.

In the Methods section, under the subheading ‘Study design’,

“This study was approved by the Research Ethics Committee Office of National Taiwan University Hospital (No. 201803013RINA).”

now reads:

“This study was approved by the Research Ethics Committee Office of National Taiwan University Hospital (No. 201803013RINA) and the Institutional Review Board (IRB) of Chang Gung Memorial Hospital (IRB No. 201800767B0C501).”

The original Article has been corrected.

